# Systematic review of global historical marine ecology reveals geographical and taxonomic research gaps and biases

**DOI:** 10.1098/rstb.2024.0279

**Published:** 2025-07-10

**Authors:** Elias del Valle, Patrick Hayes, Ilse Martínez-Candelas, Pier Brown, Loren McClenachan

**Affiliations:** ^1^ Environmental Studies, University of Victoria, Victoria, British Columbia, Canada; ^2^ Trinity Centre for Environmental Humanities, Trinity College Dublin, Dublin, Leinster, Republic of Ireland; ^3^ Department of History, University of Victoria, Victoria, British Columbia, Canada

**Keywords:** historical marine ecology, marine environmental history, systematic review, marine conservation, fisheries

## Abstract

The field of historical marine ecology (HME) developed two decades ago to address a lack of knowledge about long-term declines in the ocean. Here, we conduct, to our knowledge, the first global systematic review of HME, analysing 543 peer-reviewed articles to ask: what has been learnt and what gaps remain? The diversity of sources used in HME—from Roman texts to twentieth-century catch records—illustrates the methodological richness of the field. Most articles used documentary sources (68%) and produced quantitative outputs (54%), reflective of HME’s origins in marine science. Research focused on economically and culturally valuable taxa like fishes, which account for 41% of articles. Most research found decline (85%), while articles finding increase relied on significantly more recent data, underscoring the need for long-term data to assess decline. Strikingly, we identify geographical gaps and biases that suggest a need for targeted initiatives to support HME in the Global South. For instance, nearly as much research focused on the California Current as the entire Indian Ocean, and 74% of first authors worked in North America and Europe. Understanding the colonial legacy of marine resource extraction and the history of artefact theft that disadvantages Global South researchers should guide the future of HME.

This article is part of the theme issue ‘Shifting seas: understanding deep-time human impacts on marine ecosystems’.

## 1. Introduction

Centuries-long exploitation of marine living resources has resulted in substantial declines of many marine species globally, and modern science-based resource management has often been unsuccessful in halting these declines [1]. Understanding declines is complicated by the ‘shifting baseline syndrome’—assessment errors that occur when a modern system is compared against previous reference points that are assumed to represent the ‘natural’ or ‘baseline’ state of the system, when in fact, these baselines are also altered states [2,3]. Presented with this challenge in the late 1990s and early 2000s, fisheries scientists, ecologists and historians began to actively collaborate in interdisciplinary research seeking to understand long-term changes in marine ecosystems, for example, through the formation of the History of Marine Animal Populations group, and later, the Ocean’s Past Initiative. Interdisciplinary initiatives were furthered through working groups, such as those hosted by the US National Center for Ecological Analysis and Synthesis and the ongoing International Council for the Exploration of the Sea’s Working Group on the History of Fish and Fisheries [4]. The results of these collaborations were seminal papers [5–7] that captured international attention and became methodological foundations of the field of historical marine ecology (HME): the study of human interactions with marine ecosystems over time. Also called marine historical ecology, this field has fundamentally sought to assess cumulative impacts on marine ecosystems over time scales longer than those typically considered by marine ecologists.

Though emerging from researchers focused on marine species and ecosystems, HME is also closely related to the longer standing research field of historical ecology, which has its roots in historical geography, landscape ecology and restoration ecology [8,9]. Like historical ecology, HME has not been bound by a unified methodology and has had an applied focus [9]. While both fields are concerned with the interactions of human and natural systems in the past as they relate to modern conservation and restoration, HME derives from marine ecology and fisheries science and has focused on diagnosing and quantifying human impacts in coastal and marine ecosystems often associated with historical exploitation of marine animals. Revelations about the shifting baseline syndrome also motivated research in marine zooarchaeology [10] and palaeoecology [11,12] to reconstruct past human consumption patterns and coastal ecosystem dynamics, but these fields have their own independent and long-standing roots that rely on distinct sets of methodologies using animal remains and fossils [13]. HME also shares similarities with marine environmental history; both developed at the same time and study marine and coastal systems using similar source material, but marine environmental history has a humanities lens while HME is more rooted in the natural and social sciences [14].

HME often takes interdisciplinary approaches adapted from fisheries science, ecology, history and anthropology. As a result, it relies on a diversity of historical sources, often intended to fill data gaps and extend time series further into the past [15]. Sources commonly include those typical to social science and humanities research like oral histories [16,17] and archival documents, including maps [18,19], historical photographs [20,21], fishery logbooks [22] and historical newspapers [23]. HME research less commonly employs physical data, such as biological museum specimens [24,25].

Systematic reviews are synthesis analyses of a body of literature identified with strategic searches relevant to a given topic and are thus crucial for identifying research trends, insights, biases and gaps. They differ from traditional reviews by following a transparent and reproducible protocol that allows the results to address questions related to the field as a whole, such as identifying research gaps [26,27]. In historical ecology, a recent systematic review identified geographical and ecosystem research biases; yet this article used only data prior to 1940, which reduces its applicability to HME [28]. While there have been many reviews in HME which have collectively described sources and methods [4], synthesized results [29] and identified applied conservation value policy themes and future directions [30–33], a systematic review directed at identifying research trends and gaps has not yet been undertaken.

Here, we address this gap by conducting a systematic review that aims to provide a comprehensive overview of research trends, insights, biases and gaps in HME. Defining HME in the rich field of research approaches described above is challenging. Here, we focus on research motivation and data sources, narrowing in on HME that aims to understand long-term human interactions with and impacts on marine ecosystems using archival, oral history and museum sources. Within this set of literature, we ask the following questions:

(i) what are the publication trends in HME?
(ii) what time periods have been most studied and what data are being used?
(iii) where are HME scholars based and what areas have they studied the most? What geographical scales do HME studies cover?
(iv) what taxa have been most studied? and
(v) have HME studies identified more declines, increases or a combination of both?

## 2. Methods

We employed a systematic review protocol in this study, which included a comprehensive search for HME literature [34]. We first developed explicit inclusion and exclusion criteria on which our database searches could be based. Articles were included if they met all of the following inclusion criteria:

(i) reported on the results of original research and were published in peer-reviewed journals; review articles, books or book chapters were excluded;
(ii) biological/ecological focus (i.e. article addresses biological or ecological questions). Addressed human impact on species, ecosystems or landscapes, as opposed to primarily focusing on social or economic dimensions of history;
(iii) environmental context (i.e. article has a marine focus). Focused on coastal or marine species, ecosystems or landscapes. Articles related to anadromous species were included if they focused primarily on the marine stage of that species’ life cycle;
(iv) used archival/museum and/or oral history sources, as opposed to fossil or zooarchaeological sources. Our research aimed to identify non-traditional sources used to understand long-term change in marine ecosystems and characterize the development of this new interdisciplinary field. While zooarchaeology and palaeoecology have been fundamental to understanding such changes, they are long-standing research fields whose research methods and data have remained largely constant, requiring a specific and distinct set of expertise; and
(v) time period (i.e. article has a historical focus). Covered at least a 30-year time frame and/or included data from before 1950.

We next developed a search protocol based on these criteria, which involved several rounds of quality assessment and discussion. In a September 2023 workshop, our team identified 31 peer-reviewed research articles that clearly met our study’s inclusion criteria, but were diverse in terms of their examined taxa, ecological scale, methods, publication dates, authorship and publishing journals (see the electronic supplementary material for reference article list). We then developed a keyword search string to identify candidate articles in the Web of Science and Scopus databases, refining and improving our search terms based on their effectiveness in capturing the 31 relevant HME articles. Our final search string included five search term sets linked together with the ‘AND’ operator (see the electronic supplementary material for full search string). All our search terms were in English, which captured English language articles and non-English articles with English abstracts and/or titles. Any non-English articles were translated to English prior to screening.

Results from both searches were combined to ensure the most complete results, and duplicates were removed, leaving 3621 articles. Two researchers then independently and blindly screened these articles to confirm they met all five criteria for inclusion. In cases where both researchers agreed, the article then progressed to the next phase, data extraction. In cases where reviewers disagreed, the paper went through a second round of review by two additional researchers. Any remaining conflicts were resolved through a third round of discussion. In total, 772 articles progressed to data extraction. Articles went through a final round of vetting at this stage with 229 excluded in the data extraction phase, leaving 543 relevant articles.

Data were extracted from each article corresponding to our five main areas of investigation: (i) publication trends; (ii) sources, data and time frame; (iii) geography; (iv) taxonomy; and (v) change.

Publication trends were assessed by capturing bibliographic information about each article including authorship, publication year and journal title. We classified the articles based on their ecological study scale into three categories: species level (i.e. focused on change in single or multiple species), ecosystem level (i.e. focused on ecological interactions) and landscape-level (i.e. focused on spatial analyses).

To examine trends in sources, data and study time frames, we categorized articles based on the primary source types employed: interviews/local ecological knowledge, documentary material, museum specimens or a combination of these. We further categorized documentary material-based articles into those that employed quantitative (e.g. catch records), qualitative (e.g. newspaper articles), visual sources (e.g. maps and photographs) or a combination of these. We recorded the oldest and most recent data used and categorized each article as having either continuous time frames (e.g. time series), comparative time frames (e.g. ‘then and now’ comparisons) or intermittent time frames (e.g. patchy non-continuous data points, which often included qualitative evidence).

To look for geographical trends, we recorded the first author’s country as indicated by their first listed institutional affiliation. We also recorded the country, large marine ecosystem (LME) and ocean basin on which the articles focused. We classified articles as ‘global’ when authors specified a global scale or when articles studied locations in all ocean basins.

To look for taxonomic trends, we classified species into one of seven categories: fishes, marine mammals, sea birds, invertebrates, marine reptiles, primary producers and multiple/other, as well as 19 sub-taxonomic categories (see the electronic supplementary material for full list). Articles differed in focus, ranging from those focused on ecosystem or landscape-scale analyses which did not identify species, to those that endeavoured to assess change in dozens of species. Therefore, we classified taxa to the narrowest scale feasible, but limited species-specific data extraction to articles that studied three or fewer species.

Finally, we determined whether each article identified declines, increases, a combination of both or no change in the metric of interest (e.g. abundance, distribution or size). The designation ‘a combination of both’ refers both to single taxa whose trajectory included both decline and recovery across the period of interest and mixed results across taxa. For example, in a study focused on multiple taxa, one species may have declined while another increased.

## 3. Results

### (a) Publication trends

**Figure 1.**
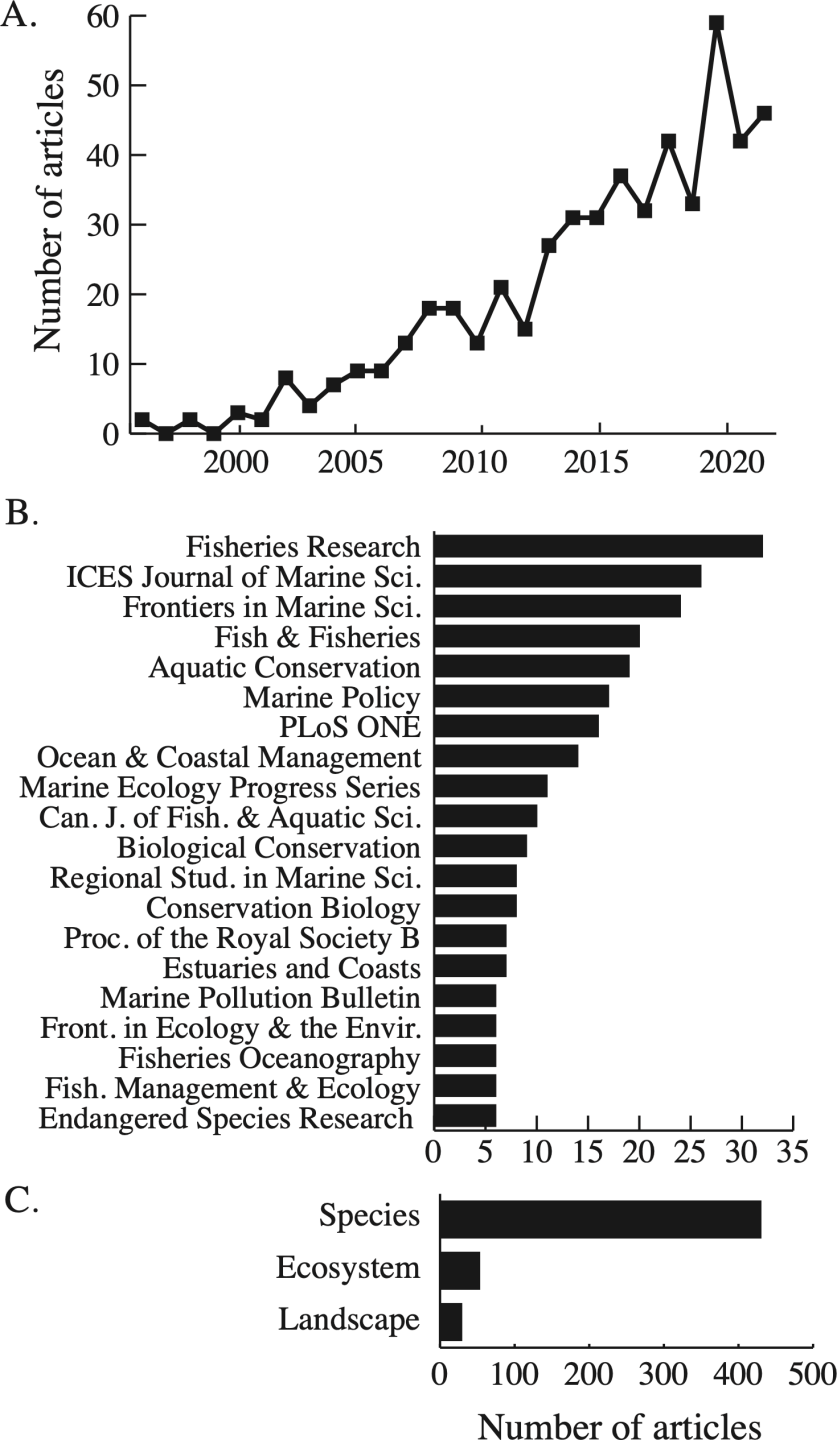
Publication trends. (A) Number of articles published annually between 1996 and 2023. (B) Journals publishing the highest number of historical marine ecology articles. (C) Ecological scale of articles.

We identified 543 HME articles published between 1996 and 2023 (figure 1A). There was a steady increase beginning in 2002 and a peak in 2020 with 59 articles. Articles were published in a range of journals, with 188 journals publishing HME research over this time scale. The top journal was *Fisheries Research*, publishing 32 articles, with 19 journals publishing more than five articles. These venues represented a mixture of fisheries, conservation and policy-focused journals (figure 1B). Most articles (79%) focused on species-level changes, and far fewer focused on ecosystem (10%) and landscape-level (5%) approaches (figure 1C). Ecosystem-scale studies often employed food web modelling approaches and landscape-scale studies typically focused on ecosystems found in coastal areas including coral reefs, salt marshes and mangroves. Another 5% used a mixture of these approaches.

### (b) Time frame, data types and sources

Most articles either employed continuous time frames (43%) or made comparisons between two time periods (38%). A smaller number (19%) had intermittent time frames. Articles with continuous time frames included both time series data, such as fisheries landings, and catch reconstructions, which often interpolate values based on a range of data types, to estimate catch over time. Articles with continuous type coverage had a median start date of 1950, as compared to 1909 for the other two data types.

**Figure 2.**
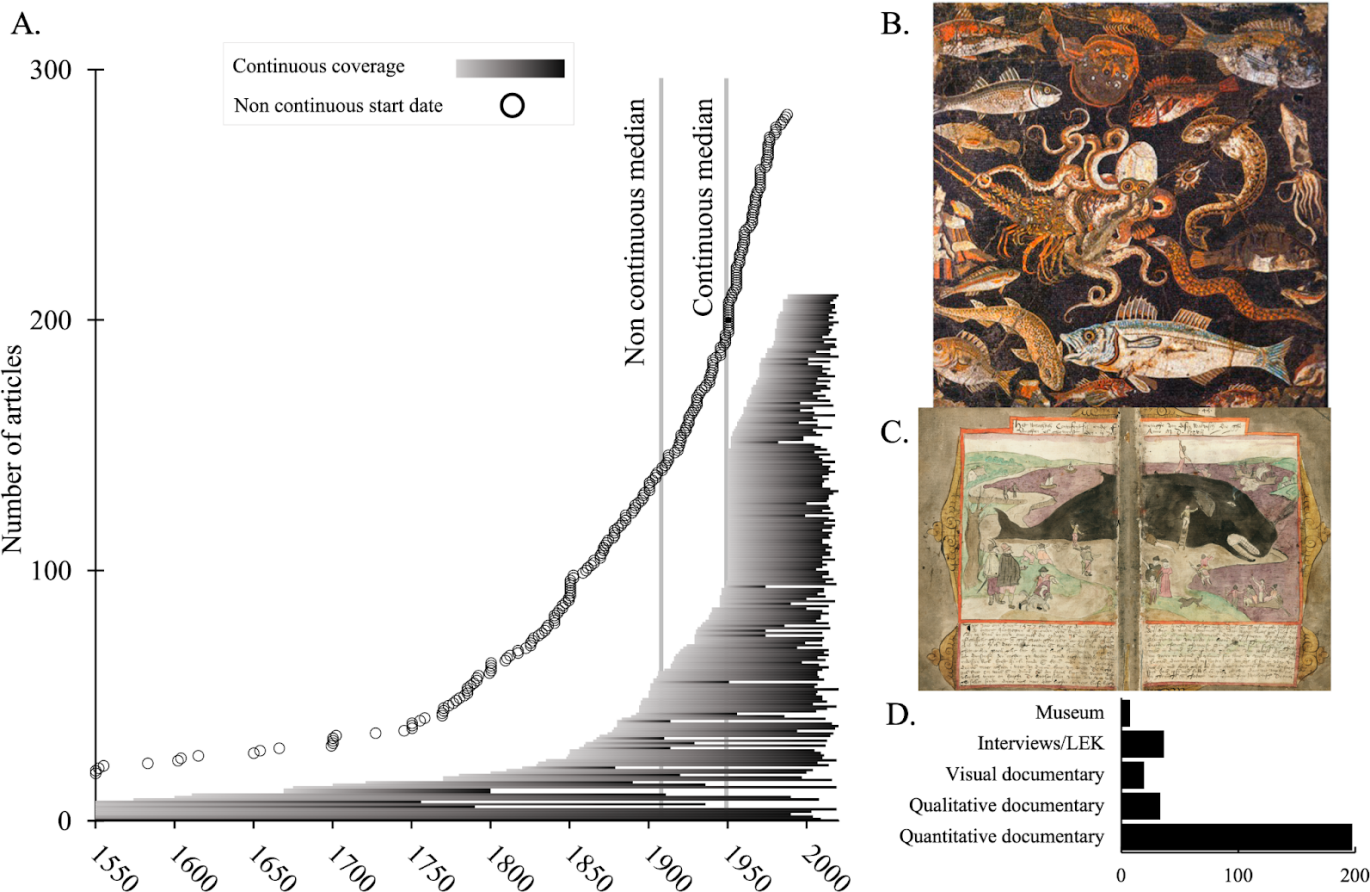
(A) Articles with continuous or non-continuous time frames and the period of time between 1550 and 2020 covered by each article. ‘Start date’ refers to the oldest data used in a given study. Note: articles with more than one time frame type (*n* = 45) are not displayed. (B) A visual documentary source from the second-century BC [35]. (C) Illustrated pages of a qualitative documentary source from the sixteenth century [37]. (D) The number of articles that used each type of source material. LEK refers to local ecological knowledge. Studies with combination source materials are omitted from the chart.

Several articles employed very old source material. For example, the article with the longest and earliest time frame documented sharks in the Mediterranean Sea over 2030 years, intermittently from 480 BCE to 1550 CE, with documentary material such as ancient Greek histories and Roman mosaics [35]. The article with the longest continuous time frame was a catch reconstruction of coral reef fisheries in Hawai’i and Florida spanning 759 years [36]. An additional 24 articles had start dates prior to 1550, 17 of which used non-continuous data and seven of which used continuous data (figure 2A).

The majority of all articles (68%) employed documentary sources. A small number (7%) employed interviews/local ecological knowledge, and only 1% used museum specimens. Approximately, a quarter (23%) used a combination of sources, with 1% employing sources that did not fit these categories. Among the articles that used documentary evidence, over half (54%) relied exclusively on quantitative sources, such as historical catch records, while 9% used only qualitative material, such as historical newspapers. We include catch reconstructions as articles using quantitative sources; this approach sometimes uses semi-quantitative sources alongside historical catch records to derive quantitative outputs, but it was not always possible to identify source material used in these analyses. A further 5% used visual documentary sources, and approximately one-third (31%) employed a combination of these types (figure 2B,C,D).

### (c) Geographical focus of research

**Figure 3.**
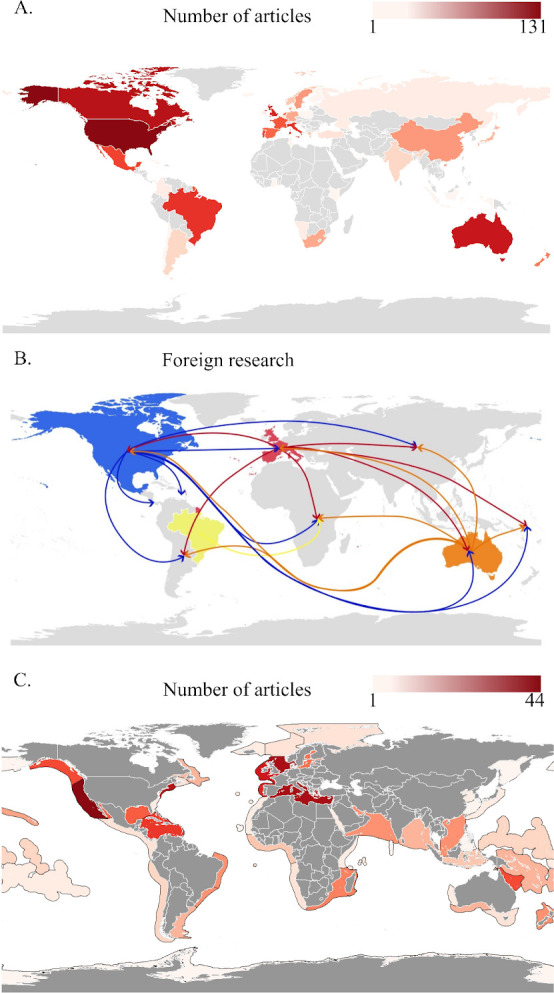
Geographical trends of research. (A) Authors’ first listed countries of affiliation. (B) A conceptual figure showing where countries with strong research output are conducting foreign research. Figure includes the 10 countries with the greatest output of HME research, coloured/grouped by continent, showing the geographical regions where they are conducting foreign research. Arrows stem from countries of first author affiliation and point towards other countries/regions where significant proportions of their foreign research is performed. (C) HME research effort at the local (LME) scale.

We found strong geographical trends in the affiliations of HME researchers, with affiliations of first authors concentrated in North America (40%) and Europe (34%), with fewer in Oceania (12%), Asia (6%), South America (5%) and Africa (3%) (figure 3A). Nearly three quarters (74%) of research was performed by researchers from just 10 countries (table 1). Articles whose first author was affiliated with a United States (US) institution produced 24% of all HME research worldwide, followed by Canada (12%), Australia (9%) and the United Kingdom (UK; 9%).

**Table 1.** The 10 countries that produced the largest number of articles, as measured by the affiliation of the first author, and the locations within which this research is performed. (Number of articles is represented as a total number and a per cent of the 543 total articles in our study. Other percentages refer to the number of articles listed for each country, with ‘within-country’ referring to the first author’s affiliated country, ‘regional’ referring to the author’s affiliated country and another country or countries, ‘foreign’ referring to a different country or countries than the country of affiliation of the first author, and ‘global’ referring to articles in which authors specified a global scale, or in which areas from all ocean basins were studied.)

country of first author’s affiliation	number of articles	within country	regional	foreign	global
United States	131 (24.1%)	62.6%	11.5%	22.9%	3.1%
Canada	67 (12.3%)	28.4%	6.0%	53.7%	11.9%
Australia	51 (9.4%)	56.9%	7.8%	27.5%	7.8%
United Kingdom	48 (8.8%)	52.1%	6.3%	37.5%	4.2%
Brazil	19 (3.5%)	89.5%	5.3%	5.3%	0%
Italy	19 (3.5%)	52.6%	36.8%	10.5%	0%
Mexico	18 (3.3%)	88.9%	5.6%	5.6%	0%
Portugal	18 (3.3%)	72.2%	11.1%	11.1%	5.6%
France	17 (3.1%)	17.6%	35.3%	41.2%	5.9%
Spain	15 (2.8%)	40%	26.7%	20.0%	13.3%

These trends in author affiliation were mirrored by the locations of research. At the continental scale, 55% of articles focused on North American and European sites, followed by Oceania (10%) and Asia (9%). At the national scale, the top countries included the US (22%), Australia (8%) and the UK (7%). In total, the UK and this small handful of countries that began as British colonies account for more than a third (37%) of all HME research output, with Canada accounting for another 5%.

While first author location largely aligned with location of research, in two countries, Canada and France, foreign research—that is, research performed outside of the country where the first author is affiliated—represented a larger percentage than domestic research. More than half (54%) of Canadian articles were focused outside of Canada, with most foreign research being done in Asia and Africa. Likewise, French and British researchers also conducted substantial research outside of their respective countries (41 and 38%, respectively) with articles focusing on overseas collectivities such as French Polynesia and former colonies like Vanuatu, the US and Australia. Two former British colonies with strong science infrastructure also participated in foreign research, with 28 and 23% of articles published by Australian and US researchers focused outside of their country respectively. By contrast, of the top 10 research-producing countries, Brazil and Mexico had the largest percentages of research within their respective countries with 90 and 89% and the smallest percentages of foreign research at 5 and 6% (figure 3B).

**Figure 4.**
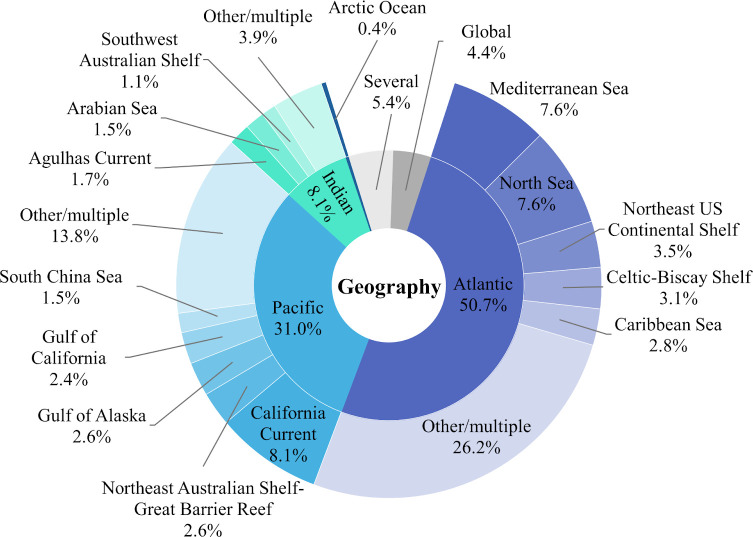
The distribution of HME research effort (*n* = 543) across ocean basins (inner ring) and large marine ecosystems (outer ring).

These geographical trends result in the historical ecology of some oceans being much more well studied than others. More than half (51%) of all articles focused on the Atlantic Ocean. The Pacific accounted for a further third (31%), followed by the Indian (10%). The smallest proportion (0.5%) of articles were focused on the Arctic. Research was further concentrated in a few LMEs. The California Current LME represented 8.1% of all articles, nearly as many as the entire Indian Ocean. The Mediterranean Sea (7.6%) and North Sea (7.6%) were the second and third most studied LMEs (figures 3C and 4).

### (d) Taxonomic focus of research

**Figure 5.**
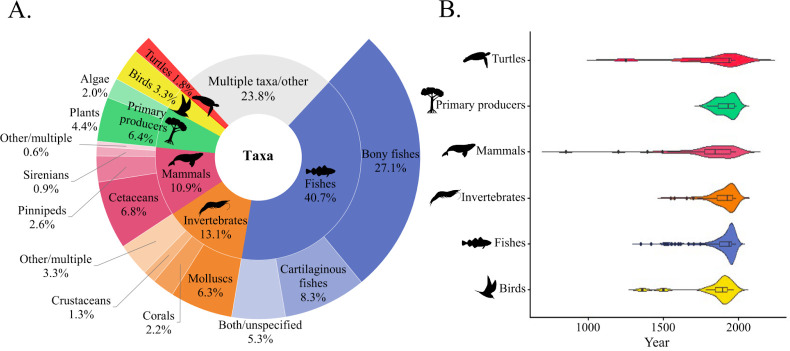
(A) The taxa represented across the 543 HME articles. The inner ring shows broader taxa categories while the outer ring shows nested contributors to these categories (if applicable). (B) Violin plot showing the distribution of oldest data used for the various taxa. Bars indicate median values; boxes indicate interquartile ranges; whiskers indicate minimum and maximum values excluding outliers and density plot indicates frequency.

Fishes were the most common taxon analysed in HME articles (40.7%), with bony fishes and cartilaginous fishes as the focus of 27.1 and 8.3% of articles, respectively (figure 5A). Articles examining fishes used data spanning a mean time frame of 118 years (including all time frame types) and had the latest median start year of 1940 (figure 5B). A few historically valuable species were well represented, with long time spans of data. For example, Atlantic cod (*Gadus morhua*) were represented in 16% of articles focused on bony fish (*n* = 23), with three articles using continuous time frames spanning more than 250 years [38–40]. Similarly, three articles that focused on Atlantic bluefin tuna (*Thunnus thynnus*) used continuous time series spanning more than 200 years [41–43].

Invertebrates were the second most frequently studied taxon (13.1%), with molluscs (6.3%), corals (2.2%) and crustaceans (1.3%) dominating this category. Among articles that examined three or fewer species, European flat oysters (*Ostrea edulis*; *n* = 9) were the most commonly analysed taxon. Articles examining invertebrates used time series that spanned a mean of 109 years and had a median start year of 1925. The longest continuous time frame was a catch reconstruction of rock lobster (*Jasus frontalis*) fisheries in Chile that spanned four centuries [44].

Mammals were the focus of 10.9% of articles, with most researching cetaceans (6.8%), followed by pinnipeds (2.6%) and sirenians (0.9%). Among articles that studied three or fewer species, sperm whales (*Physeter macrocephalus*) were the most frequently studied marine mammal species (*n* = 10 articles). Articles focused on marine mammals had a mean time frame of 175 years and the earliest median start year of 1845.

Primary producers were studied in 6.4% of articles, with plants and algae at 4.4 and 2.0%, respectively. Seagrasses including dwarf eelgrass (*Zostera noltii; n* = 2) and smooth cordgrass (*Spartina alterniflora; n* = 2) were the most researched taxon in articles that examined three or fewer species. Articles examining primary producers had the shortest mean time frames, at 100 years and a median start year of 1933.

Birds were the focal taxa of 3.3% of articles. Among articles that examined three or fewer species, Glaucous-winged gulls (*Larus glaucescens*) were the seabird species most commonly studied (*n* = 2). Articles researching sea birds had the longest mean time frame at 235 years, with a median start year of 1896. The longest article on long-term changes in bird habitat in the Netherlands covered a period of 1965 years [45].

Marine reptiles were the focus in only 1.8% of articles (*n* = 10). Among them, green turtles (*Chelonia mydas*) were the most researched species, featuring in nine studies. Articles researching reptiles had the second longest mean time frame at 179 years and had a median start year of 1938.

### (e) Change found

**Figure 6.**
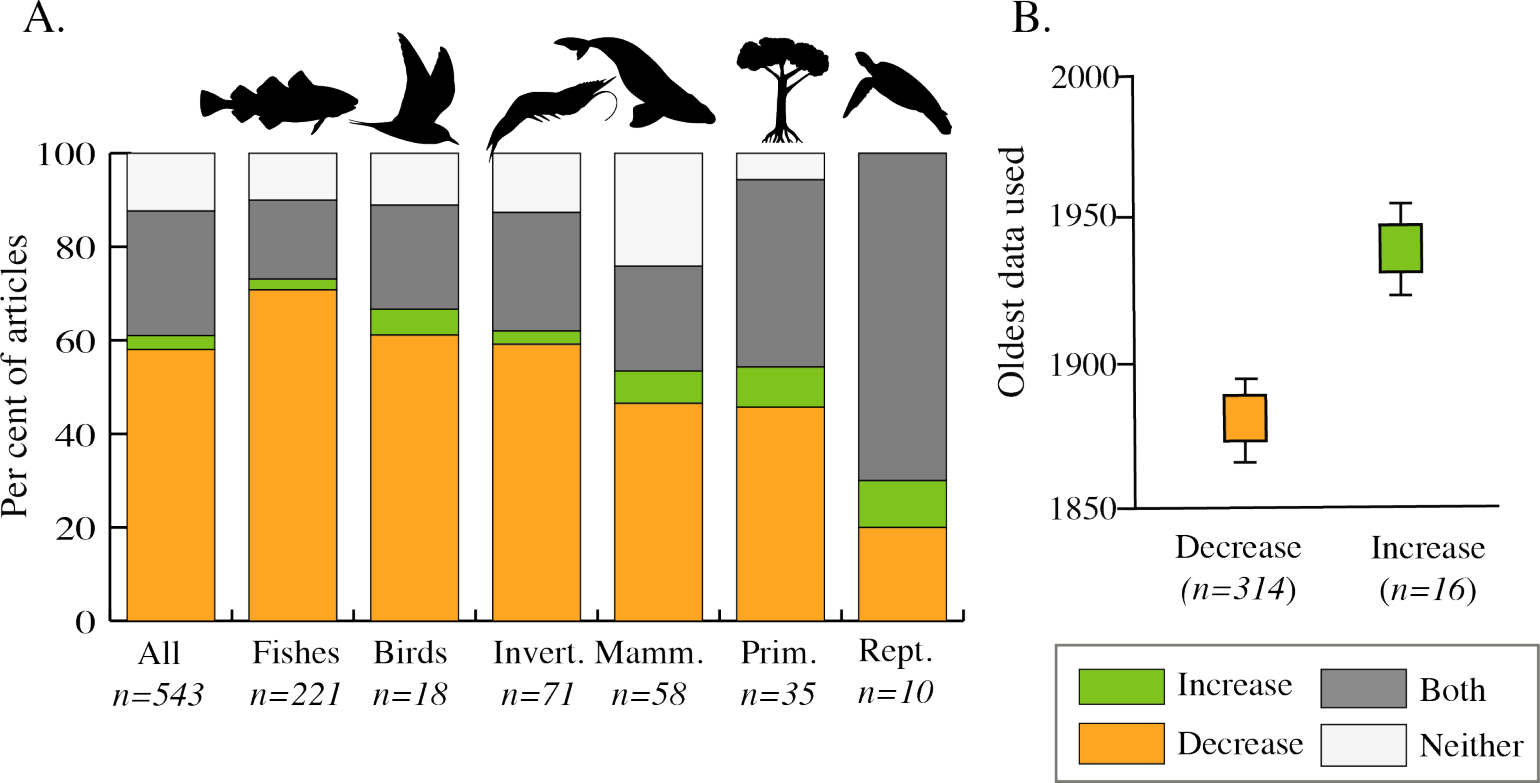
(A) Change found across six taxonomic categories, with studies finding increase (green), decrease (orange), both increase and decrease (grey) and neither increase nor decrease (white). (B) Oldest data used for studies finding increase and decrease; studies finding decrease used older data (mean date of oldest data 1873, s.e. 8.40, range 850−1988) than studies finding increase (mean date of oldest data 1942, s.e. 9.47, range 1835−1986). Values represent means and standard errors. Differences are significant at the *p* < 0.05 level.

Collectively, articles demonstrate declines across most taxonomic groups (figure 6A). Most articles (58%) found only decline, while far fewer (3%) found only increase. Approximately one quarter (27%) found a combination of decline and increase, while 12% found neither. Articles that found a combination of decline and increase included those that found recovery following decline, as well as evidence of decline in some focal taxa with increase in others. For example, one article using fishers’ local ecological knowledge reported changes in the relative abundance of target fish species in the Mediterranean with some species increasing and others decreasing [46]. Another identified an increase in green turtle abundance in the past few decades following historical declines over centuries [47].

Across taxa, fishes saw the greatest frequency of decrease with 71% of articles finding decline only, followed by birds (61%) and invertebrates (59%). By contrast, reptiles and primary producers demonstrated the highest frequency of increase only (10 and 9%, respectively), as well as a combination of decline and increase (70 and 40%, respectively). As noted above, the long-term decline and recent partial recovery of green sea turtles—the most studied reptile—accounts for this pattern in reptiles. Similarly, in primary producers, combined declines and increases were exemplified by cases such as eelgrass, where human developments have impacted substrate and resulted in localized declines and recoveries through time [48].

One half (47%) of articles on marine mammals found decline only, with 7% finding increase only and 22% demonstrating both. Like green turtles, many marine mammals have a history of exploitation over centuries, with partial recovery since protections in the late twentieth century, which accounts for this pattern. Nearly a quarter (24%) of articles on marine mammals found neither decrease nor increase, which was often because of the use of strandings time-series data in studies that were not designed to assess change. The inclusion of these articles highlights the nature of systematic review papers; while these studies met our inclusion criteria in the use of long-term (30 years+) time series, their aim differed from most studies in HME.

The duration of data affected findings of increase or decrease (figure 6B). Across taxa, the few articles finding only increase used more recent data than the many more articles finding only decrease, with significant differences in mean time frame start years (Welch’s *t*‐test, *t*_44.40_ = −5.36, *p* < 0.001). The 314 articles finding decline only had a mean start year of data of 1873 (s.d. = 149.34), while the 16 finding increase only had a mean start year of 1942 (s.d. = 39.05).

## 4. Discussion and conclusion

HME researchers have been innovative in their approaches to understanding past marine ecosystems. Our results showcase the great variety of sources used in HME articles, ranging from ancient Roman mosaics to early modern ships' logbooks, interviews with local knowledge holders and annual reports of fisheries catch. HME researchers are most commonly using quantitative documentary evidence, with one commonly employed approach being catch reconstructions. Spearheaded by the Sea Around Us—a University of British Columbia based research initiative—catch reconstructions had the goal of more fully accounting for fisheries catch since 1950, when the Food and Agriculture Organization began producing annual global fisheries reports, which are often incomplete owing to a reliance on national-level reporting [49,50]. Catch reconstructions employ a diversity of data types to fill in these data gaps, but the output is quantitative in nature, which is also reflective of the majority of HME research we reviewed. While HME is characterized by diverse methodology, a focus on quantitative outputs is reflective of the nature of the research questions being posed by researchers in the field. It is notable that qualitative data are often used in support of these goals, but the comparative lack of HME research using qualitative sources may also be reflective of a reluctance to accept qualitative data as a robust source of information on the oceans' past.

Our review illustrates that global marine species decline over century-long time scales is pervasive, with fishes, birds and invertebrates being the taxonomic groups where the greatest numbers of articles reported decline. Articles that identified only decline used data with significantly older start dates on average, supporting the idea that the duration of data has an impact on the type and magnitude of change identified [32,51]. Using long-term data is therefore critical to informing species and ecosystem recovery targets, as employing more recent data alone may underestimate ecological potential and result in recovery targets set too low [32]. Long-term data are also needed to place recent recoveries of marine species into the longer history of decline [51]. Despite this overall trend of decline, one quarter of articles found both decline and increase, supporting observations that marine species, such as turtles and whales, are recovering after historical declines [32,51].

Our results show that HME research is biased towards certain taxa. This was no more evident than in the disproportionate study of commercially important fish and shellfish such as Atlantic cod and European flat oysters, and charismatic marine fauna like sperm whales and green turtles. An emphasis on such species highlights the economic and cultural value that certain taxa have held for humanity at different points in history. While there may be an element of modern bias in research preference and funding, this trend also probably reflects historical observational bias—that is, a greater extent of available historical data for species that have historically been economically and culturally important [15]. These are also often the taxa that have experienced decline owing to targeted exploitation, which makes them important species to investigate. Nonetheless, we recommend that future HME research efforts engage with under-represented taxa, and taxa whose historical decline may be the result of by-catch, habitat destruction or other impacts for which historical records are less available [52]. In these efforts, collaborative work with archaeologists, palaeontologists and others whose sources are not biased towards the same taxa is critical [12].

We also identified significant geographical gaps in HME research. For instance, surprisingly little research was focused on Indian (10%), Arctic (0.5%) and Southern (0%) ocean basins. While data availability has undoubtedly influenced this trend, future research should endeavour to address this gap, particularly as the resilience of both tropical and polar ecosystems is threatened by the synergistic effects of climate change and overfishing [53,54]. Likewise, at the LME scale, a large proportion of research effort is concentrated in ecoregions that border Europe or the US, leaving many LMEs with little to no historical ecology research. These gaps mean that HME research may be overlooking threatened marine biodiversity hotspots, such as those in Southeast Asia [55]. Together with our finding that researchers are concentrated in North American and European institutions, these findings suggest that a key next phase in HME is to support the development of research capacity outside of North America and Europe.

Geographical biases also exist that affect the generalizability of research results and suggest a need for targeted initiatives supporting equity in HME research. It is particularly notable that more than one-third (35%) of all articles were focused on former British colonies (i.e. US, Canada and Australia). These patterns mirror those in the field of historical ecology more broadly [28,56]. Importantly, these locations have a unique but shared legacy of colonial resource exploitation, thus deriving generalizable trends from them may be inherently problematic. Colonial resource exploitation, of course, was not unique to Britain. Dutch, French, Portuguese and Spanish colonization mobilized extensive extraction of not only natural resources but also of cultural, artistic and natural history artefacts of high relevance to HME. Many of these remain in European institutions to this day [57]. As such, Global South researchers are comparatively disadvantaged in accessing historical ecological data of relevance to their own regions. This dynamic has also contributed to ‘parachute science’—that is, Global North researchers leading research endeavours in the Global South—a widespread trend in conservation science [58], with implications for career progression of Global South researchers as well as capacity building of local institutions [59]. Our findings reaffirm the continued need to support Global South scholars, with strategies that include increasing research referencing and recognition, reducing open-access publication costs, increasing research group diversity, as well as digitizing archive and museum collections to increase accessibility [60,61].

Our study is characterized by several limitations. First, our search query was limited to peer-reviewed articles with titles, keywords and abstracts in English, which may have overlooked non-English articles and contributions published in books or grey literature publications from authors in the Global South [56,62]. Moreover, because we only recorded the first listed affiliation of first authors, it is likely that some collaborative research endeavours between Global North and South partners may have gone unrecorded. Owing to the impossibility of tracing first authors’ individual academic trajectories or life histories, we may also have grouped Global South scholars studying or working in Global North institutions as part of ‘parachute science’. However, these methodological limitations do not take away from the general trends that reflect unequal opportunities in the HME field. Second, owing to the nature of systematic review protocols (e.g. the specificity of search terms developed in support of the inclusion criteria), some relevant HME articles will not have been captured. For example, we are aware of HME research focused on saltwater crocodiles that was not captured by our search terms [63]. Therefore, despite efforts for taxonomic inclusivity, our search terms may have been biased. While designed to be objective and comprehensive, systematic reviews can omit studies that use alternative terminology or that fall outside of predefined categories.

In conclusion, this first, to our knowledge, systematic review of the growing field of HME synthesized a large and diverse body of research, with more than 500 peer-reviewed articles from around the world. The diversity of sources used in HME research—from ancient texts and oral histories to modern catch reconstructions—illustrates the methodological richness of the field, which not only enhances the robustness of findings but also underscores the importance of integrating historical data with contemporary ecological research to inform sustainable management practices. As HME evolves, its tools remain pivotal in documenting the complex interactions between human societies and marine ecosystems throughout history to inform modern conservation. Taken together, our review highlights the need for future HME research to prioritize under-represented regions, including the Arctic, Southern and Indian Ocean basins, as well as biodiversity hotspots, such as those in Southeast Asia. Additionally, HME studies should focus on less ‘charismatic’ species and those whose declines may not have been recorded in the sources commonly used in the field, such as catch records. Finally, the field must place greater emphasis on supporting and amplifying research led by scholars from the Global South. Such efforts will further strengthen the applicability of HME research to contemporary marine conservation and restoration efforts globally.

## Data Availability

All data are available in the electronic supplementary material [64].
